# Relational, Appreciative, and Process-oriented Digital Storytelling: A Duoethnography

**DOI:** 10.1007/s42087-023-00337-7

**Published:** 2023-03-14

**Authors:** Alexios Brailas, Chara Sotiropoulou

**Affiliations:** 1 Department of Psychology, Panteion University, Athens, Greece; 2 Athenian Institute of Anthropos, Athens, Greece

**Keywords:** Digital storytelling, Appreciative inquiry, Narrative practice, Systems thinking, Duoethnography, Group work

## Abstract

In this paper, we propose an approach to group and community digital storytelling based on the following pillars: (a) extensive periods dedicated to group forming; facilitating meaningful interactions and members synergies through organized group work activities, (b) cultivating an appreciative and inclusive culture that takes advantage of the individual differences, and (c) a process-oriented practice, informed by systems thinking, that listen to the individuals’ voices and needs as well as to the group-as-a-whole process. In this context, digital storytelling could help people and communities to re-author themselves in more empowering, meaningful, and life-promoting ways. In the first part of this work, we discuss the theoretical underpinnings of our approach, we describe the details of a first pilot offering, and we provide an initial narrative evaluation by the participants. In the second part, we engage in an evocative duoethnography of our practice to provide a narrative, performative, in-context, and dense description of our lived experience as designers and facilitators, and as human beings that we actively participated in the unfolding of our program. Implications for the transformative power of duoethnography are discussed.

## Introduction

In this paper, we propose a narrative intervention program that combines digital storytelling with facilitated group work aiming to build an appreciative space for personal and collective development within a culture of inclusivity, and we study its first pilot delivery through an evocative duoethnography. In the specific digital storytelling program, the emphasis is not on the *digital*. Digital tools are only used to help participants engage in a multivoiced, multimodal, relational, and collaborative modern community storytelling practice. A great challenge in community work is to give voice to minorities and vulnerable groups and to create dialogic bridges between insiders and outsiders, scholars, and research “subjects.” In the context of the “giving voice,” participatory, community-based, research paradigm, digital storytelling (DST) can be used as an active way of realizing, capturing, and sharing participants’ untold stories. In the action research tradition, DST can also be used as a way to create storytelling communities and, by doing so, empower people and develop networks of resilience (Brailas, 2021; Papadopoulos, 1999).

DST offers an opportunity for rich multimodal media production by communicating the voices of the participants in a way that is less likely to happen through textual-only narratives (Jamissen, 2017). The diverse modalities employed in DST facilitate a multilayered cognitive processing (informational, emotional, and cultural) (Alonso et al., 2013). Especially in a community/group context, DST experiential workshops can create a sense of empathy and understanding, which allows participants to enrich their personal narratives by recognizing and incorporating different perspectives (Glesne & Pugach, 2018; Lambert, 2013). The process of developing authentic life stories in a digital multimedia format helps people realize their untold, enabling or constraining, narratives. By sharing their stories people create meaningful spaces for collective empowerment: “These narratives possess the capacity to inhibit an individual’s growth and happiness, or can empower and enable wellbeing and contentment” (Casares & Gladding, 2019, p. 3). It is through the reflective process of narrating and (re)authoring themselves that people develop and enrich their own identities.

Participants in DST programs interact with their digital stories during their development as well as during their distribution and sharing, resulting in multilayered reflective processing of personal experiences. Nurturing an appreciative inclusive culture allow participants to feel safe to share their stories (Brailas, 2021). In the context of an appreciative community, DST can provide a welcoming inclusive space to discuss stigmatized topics, and give voice to marginalized groups (Gachago & Livingston, 2020; Guse et al., 2013; Matthews & Sunderland, 2017). Storytelling combined with group work enables participants to support each other in addressing prior trauma and reauthoring stigmatizing personal narratives (Gubrium et al., 2019). Stories effectively communicate critical voices and different perspectives (Glesne & Pugach, 2018, p. 31).

Facilitated group work and organized peer-to-peer interactions within an appreciative culture engage participants in a group forming process where they feel safe to share their stories and where they can offer and receive valuable feedback. In the virtual mirror that is created through the story sharing, it becomes possible for the participants to recognize their own unspoken voices and hidden narratives (Brailas, 2021). In terms of the Bakhtinian theory, this is “the tale of how I get myself from the other” (Holquist, 2002, p. 28). However, the benefits of the group feedback provided to each participant are not restricted only to the informational and cognitive surplus that will ultimately enrich their stories and personal narratives. It is through the group work and the resulting peer-to-peer interactions that participants “practice how to relate and connect with other human beings, and how to create meaningful personal networks.” (Brailas, 2021, p. 12) And this skill lies at the core of a culture of inclusivity: nurturing the ability to relate and connect instead of isolating, scapegoating, or attacking the different other. This intra- and inter-personal work is a prerequisite for promoting social-emotional learning and raising empathy (Brailas, 2020; Goleman & Senge, 2014).

Through this work, we attempt to provide a rich multimodal description of the implemented program and our lived experience as designers, facilitators, humans, and, finally, as co-creators and co-participants in an appreciative storytelling community. As Von Foerster (1984) points out, “The way in which a question is asked determines the way in which an answer may be found.” (p. 46) The purpose of this article is to present the specific community digital storytelling program as a living curriculum, as a form of *currere* (Paul & Beierling, 2017). Currere in curriculum studies stands for a conception of the curriculum as a multiplicity that is always in the becoming: a living process rooted in the past, experienced in the present time, while being oriented in the future (Pinar, 2019). In the first part of this article, we present our community digital storytelling program in a more static curriculum format: we provide its rationale, design, and the details of its first delivery and evaluation. In the second part of this article, we continue with a duoethnography of our praxis as a way to present the program in a more dynamic, convivial, and transformative format. By doing so, “the act of recalling meaningful events and reading personal beliefs within a playful yet disciplined dialogic frame itself becomes part of the currere and subsequently the duoethnography.” (Sawyer & Norris, 2013, p. 15) The two parts of this article are intertwined and complement each other.

## From Storytelling to Digital Storytelling

Storytelling is the process of creating and sharing stories in many forms, like fairy tales, myths, legends, (auto)biographies, or anecdotes. Every human culture develops its unique techniques and tools to use in the storytelling process. In the recent years preceding the rise of the Internet, people employed mainly photo and video cameras for visual-enhanced storytelling production. Today, digital multimodal stories can be produced easily by anyone without the need for special technical skills. For example, modern teens constantly produce storytelling artifacts, usually very short stories in various visual formats, in today’s social media platforms and apps.

By using *Google’s Ngram viewer*, a tool that detects word sequences throughout the digitized corpus of books, we observe that the term *storytelling* has been increasingly used more and more since the 80s and afterward. As regarding the combined term *digital storytelling,* there is a dramatic, almost exponential, increase in the use of the term since 2004, with the increase beginning just after the middle of the 90s. A time that coincides with the rise of web 2.0 technologies and social media. It seems that digital social media serve an inherent need of people to tell stories, to storify their existence. Why are we fascinated by storytelling? Is it an emotional call, the search for a magic place and time, or the need for creating an alternative reality? Does remind us of our lost childhood? We were little kids then and we used to listen to fairy tales. Do adults use digital storytelling as a desire to return to this lost Eden Garden? Is it an archetypal human activity? Maybe it is a fundamental human need to share our stories, to leave a trace behind: *I was here, and this is my story…* In the end, maybe we are just *storytelling animals*:

Humans are pattern-seeking, storytelling animals. We look for and find patterns in our world and in our lives, then weave narratives around those patterns to bring them to life and give them meaning. Such is the stuff of which myth, religion, history, and science are made. (Shermer, 2000)

Regarding digital storytelling, a major opportunity that this specific genre presents is that digital stories are concrete artifacts with a material presence, in a video, comic, or other digital media form. So, they can be easily viewed and reviewed, shared face-to-face or online, performed and re-performed, modified, and fine-tuned. In a social constructionist and appreciative inquiry tradition, “words” matter, and the stories we tell do not just describe the world within and around us; our stories produce that world. The stories we tell bear the power to make us laugh, make us cry, move us forward, or inhibit our growth. Literally, by reshaping our (digital) stories, we can reshape and further develop ourselves.

## Community and Storytelling

Human communities are complex social systems that develop, change, and transform to adapt to the evolving conditions of their social and cultural context (Schön, 1971). In English, the term community comes from the Latin words *com* (meaning together) and *unus* (the number 1, the unit) (Delanty, 2010). Therefore, from an etymological point of view, the concept of community can be realized as the social whole created by the individuals when coming together. The community symbolizes a paradise lost and, at the same time, a paradise to which people wish to return (Bauman, 2001). As Delanty (2010) points out, “the popularity of community today can be seen as a response to the crisis in solidarity and belonging that has been exacerbated and at the same time induced by globalization.” (p. x). The term community seems to resemble a Rorschach blot upon which people project their hopes for a better future (Kirp, 2001). Often the term is used without giving any specific definition, implying that the concept falls within the realm of common sense, is automatically and universally understood, and therefore does not need to be defined (Creed, 2006). It is characteristic how difficult is to use the concept of community with a negative connotation.

Hillery (1955) conducted a content analysis of ninety-four different sociological definitions of community and identified three basic conceptual components: interaction, common ties, and a common space for action (Hillery, 1955). Community members are constantly transformed through their participation. At the same time, members are constantly negotiating and developing a shared community culture. This is a form of mutual determinism: the members, through their cooperative action, create their community, and at the same time the emergent community shapes its members (Baranowski, 1990). Communication problems can inhibit the development of a community. Communication is so important that in the past spatial proximity was a prerequisite for the development of a community. Today, information and communication technologies facilitate the formation and development of communities without the need for physical proximity. The members of a community usually share common experiences, common meanings, and a common culture, while they negotiate shared visions of the future (Sanders, 1966). The community seems to satisfy the basic human need for belonging to a group and being recognized by others.

In this article, we define as a community a group of people who engage in dense and personally meaningful interactions constantly negotiating a shared purpose. Storytelling creates community, a place to belong (Lambert, 2013): an appreciative space to share your intimate thoughts, your achievements or your vulnerabilities. Or is it the other way around and it is the community that creates its stories? (Papadopoulos, 1999) What comes first? What we have learned from the first offering of our program is that digital storytelling in a community context and within an appreciative and inclusive culture bears the potential to move us to a new place where we feel safer to share and exist, in an *I share therefore I am* mode of being. It seems that communities create stories and stories create communities in a circular process of mutual determination. Having these considerations in mind, in our digital storytelling program we paid special focus on facilitating group forming activities and nurturing an appreciative group culture.

## The Program *Digital Storytelling Lab: Stories That Make Us More Humane*

The specific DST program entitled *Systemic Digital Storytelling Lab: Stories that make us more Humane* aimed to help participants realize alternative, more polyphonic, ways of acting, thinking, and relating with each other. The program was offered by the Athenian Institute of Anthropos (AIA), a nonprofit organization for the study, research, training, and development of applications for systemic practice, psychotherapy, and personal development, located in Athens, Greece. The workshop aimed to empower the participants through the creation and sharing of digital stories. Through the workshop, participants became familiar with this community, process-oriented, and appreciative approach to digital storytelling, to utilize it in their practice with individuals and groups. Meanwhile, they familiarize themselves with the necessary tools for digital media production, (re)discovering their creative potential.

The program was aimed at educators, psychologists, social workers, and, in general, practitioners working with human systems and communities. The program lasted twelve weeks, from January to April 2021. In the twelve weekly 2-h meetings, participants learned how to create their digital stories, how to share them, and how to offer and get constructive peer feedback aiming to rewrite and produce more enriching versions. The digital stories produced became the mediating tools for personal and collective empowerment in the context of an emerging appreciative and inclusive group culture. The short description that was shared on social media and through emails to promote the program follows:

The *Digital Storytelling Lab: Stories that make us more humane* is a distant (due to COVID-19 restrictions) learning program that aims to empower participants through the creation and sharing of personal stories by using digital media. The personal transformative process of creating digital stories allows us to unearth suppressed personal voices while acknowledging the dominant ones that drive our actions, to integrate them into a richer and more polyphonic ensemble. In addition, appreciative group work contributes to the development of a safe place to work and develop participants’ stories and a trustful audience that will listen to individual narratives and provide enriching feedback.

During this program, participants were asked to create a short storytelling artifact following each weekly synchronous meeting, reflecting on their lived experience during that session, and telling their own story about this. Some guiding questions were offered as a prompt for their storytelling: *what I understood*, *how I felt, what I like to ask, and how can I utilize this in my professional practice or personal life.* Every week, a part of the meeting was dedicated to training in digital tools and media platforms suitable for storytelling. Therefore, weekly storytelling artifacts became gradually more enhanced with extra modalities and media. Meanwhile, during the 4th meeting of the program, we presented participants with a specific theme for their final, and more complete, digital stories. From our previous experience with digital storytelling workshops in various settings, we knew that an umbrella theme helps the group members to stay focused and develop their stories. In the *Stories that make us more humane* program, we chose the following umbrella theme for the concluding storytelling: “Share through a digital story the details of your personal relationship with your profession, in your long personal time: how it all started, where is now, and how you are dreaming it to become in the near and far future?” In an appreciative inquiry tradition, words are not neutral; they create our reality. Appreciative inquiry recognizes the power of the words and, therefore, the power of the questions we ask to create the future we long for (Bushe, 2012; McAdam & Lang, 2003). So, in the umbrella theme we chose, we impart an appreciative inquiry element, that of storifying how somebody dreams to be in the future, as an inquiry into the desired potential of the individuals and their community. Dreams of a desired future become “a generative and collaborative process where people, in relationship, join to discover abilities and values in these treasured episodes and dream of a future in which we live the values behind the abilities.” (McAdam & Mirza, 2009, p. 176).

As designers and facilitators of this program, we the authors took care to create digital stories all the time to communicate any information needed or reflect back to the group. So, we acted in an isomorphic way to what was asked by the participants to perform (Brailas, 2017). In the same direction, when we needed to present our work at a conference (TAOS Institute conference, *Education as relating*, November 4–6, 2021), we opted for creating a digital story-style version of our presentation, effectively storytelling our own practice. This digital story that provides a multimodal introduction to the *Digital Storytelling Lab: Stories that make us more humane program*, can be accessed at the following link: https://www.youtube.com/watch?v=wK59_I9L4o8.

## Nurturing Community: Group Work

In our approach, we realized that to nurture an appreciative storytelling community we need first to create a cohesive group. In every single session of our twelve meetings program, a substantial part of the time was dedicated to group work activities aiming to foster meaningful connections among the participants. In this way, we aimed for the short digital stories produced by the individuals following every week’s meeting to be the byproduct of a group process. Community building requires effort and takes time, and a coherent group can be the ideal environment for the emergence of inspiring storytelling circles. By using the term coherent group, we mean a group of people that starts to work as a whole. Therefore, it is able to demonstrate emergent properties as the byproduct of members’ synergies. Emergent properties cannot be reduced to any linear sum of the individuals’ properties. It is what the group-as-a-whole can achieve that cannot be achieved by simply summing up individuals’ efforts. As Agazarian and Gantt (2000) point out, “the group-as-a-whole increases the group’s ability to discriminate and integrate differences and that systems development and transformation occurs through the integration of different communications.” (p. 68).

In such an approach, the facilitators’ role is to help participants realize new perspectives, alternative connections, and new patterns of relating with each other, and with their digital storytelling artifacts. The community as a whole becomes the apparatus for both personal and collective empowerment, a multiplier of perspectives, and an amplifier of synergies (Brailas, 2021). As Michael Bakhtin points out, “Truth is not born nor is it to be found inside the head of an individual person, it is born *between people* collectively searching for truth, in the process of their dialogic interaction.” (Bakhtin, 1984, p. 110). We suggest that this kind of storytelling community creates the background and the meaningful context for developing empowering individual and collective narratives.

## Stories to Make Us More Humane

Digital storytelling can be realized as experiential learning, a form of learning by doing, learning by creating digital storytelling artifacts with a concrete material presence. By changing those digital stories, we change our own, empowering or inhibiting, narratives. The different possible ways of being and relating with each other realized during the group interactions can enrich the new story versions produced. In the process, the group shapes the individuals while as a whole is shaped by members' actions and synergies. What one can be in a group depends on the group as far as the same communication may elicit different responses from the group at different points in time. Nevertheless, “one is completely responsible for what one does not say in a group, in that the group does not have the opportunity of addressing the information.” (Agazarian & Gantt, 2000, p. 59). In the virtual mirror that is created through the sharing of all the group stories, each participant can recognize her own unspoken voices and hidden narratives (Brailas, 2021). In other words, we co-author ourselves in the (back)ground that we co-create by sharing our stories. Or in the words of Cozolino (2014):

Relationships are our natural habitat. We need each other and our stories to discover ourselves, regulate our emotions, and heal from traumatic experiences. Humans serve as external neural circuits that we can use to help each other bridge dissociated neural networks, provide us with new ideas, and get in touch with feelings that we may be unable to access or have forgotten to remember. When loving others link their brain with ours, the result is a vital integration. We can use our interpersonal resonance, intuition, and empathic abilities to help and heal one another. Human brains have vulnerabilities and weaknesses that only other brains are capable of mending. (p. 393).

The others become external positions in the repertoire of different voices of the self (Hermans, 2001), also operating as an appreciative audience, and as appreciative witnesses that reflect and promote different modes of experience. While shaping and re-shaping the story artifacts, on the level of the self, reflections, conflicts, and negotiations may arise (dominant vs. less dominant positions vs. emergent positions, etc.) offering more awareness and enrichment in an ongoing dialogue that takes place in different levels (intra-personal and inter-personal). Effectively, it is not only the group space but also the personal intra-spaces that are reshaped through interaction.

The story “circle” becomes a transformative ritual within the group that in this context can validate the shared experiences. At the same time, the whole group builds its own story and shapes its world, it storifies its collective existence. Individual stories become manifestations at a personal level of the diversity of voices that are present at the whole group level. The story artifacts become statements, processes in an ongoing dialogue. A digital story artifact is not the end of the storytelling process; it is only the beginning of a new circle contributing to an ongoing transformative process of the self and the group.

## Evaluation of the First Offering of the Program

Twelve people participated in the first offering of our program. In the concluding meeting, we invited all participants to provide a short evaluation of the program and ten participants responded with their reflections on the question: *What does this program (the Digital Storytelling Lab: Stories that make us more humane) mean to you?* We conducted a thematic analysis (Campbell et al., 2021) on these reflections (Appendix), and we identified three central themes, which we discuss in this section.

### Theme A: Connecting/Relating with Each Other

Making and creating stories in the context of an emerging community seems to facilitate creating connections and relating with each other: “This is about creating a connection with own self and with the group” (participant 1), “it is relating to myself, to the others, and to the outer world” (participant 2), “a way of sharing, connecting, and telling stories” (participant 4). “The group became a safe place to rest upon and share” (participant 6). “It is relating. This is the first spontaneous answer that comes to me. I could stop writing here” (participant 7). “Making and sharing stories that connect people” (participant 9). “Medium for expression, for communication, for relating with each other … the magic of connection” (participant 10). By facilitating human connections and interrelations, this relational, appreciative, and process-oriented approach to digital storytelling seems to help develop a sense of community for people.

### Theme B: Creativity/Expressing/Sharing

Participants in their reflections focused also on some affordances of community digital storytelling such as nurturing creativity and the ability to express themselves and share: “It is a way of expressing feelings … a way of sharing … Technology and people are coupled to create” (participant 1). “Creativity, co-creation, and exploration” (participant 2). “My creative self, my writing self, my determined self” (participant 7), “The use of colors and sounds, drawings, animation, and other creativity tools” (participant 9). “When we manage all together to create a whole, the beauty of sharing becomes magic” (participant 10).

### Theme C: Self-development

Finally, participants also focused on aspects of self-development through their collective storytelling practice: “A kind of memory recording that helps you observe your evolution, what was your beginning, and where you have arrived” (participant 1). “It helped me to rediscover sides of myself that I had forgotten long before” (participant 2), that “creates memories, and lived experiences, on a collective or individual level” (participant 5), “an oasis during the week” (participant 6). “I also reconnected with aspects of myself that for some reason I neglected to bring to the foreground” (participant 7).

## Duoethnography: Autoethnographies in a Transformative Dialogue

### The Tenets of Duoethnography

Sawyer and Norris (2013) identified a set of twelve tenets for duoethnography we discuss in this paragraph. These tenets are not proposed as unquestionable axioms but rather as the living pillars of an evolving research practice. The first tenet, *currere*, is the realization of duoethnography as a living dialectical process aiming for participants to gain new knowledge on existing phenomena: “The goal of a dialectical interaction is not a greater understanding of existing meanings and interpretations. Rather, it is the reconceptualization of those meanings.” (Sawyer & Norris, 2013, p. 15) In the Bakhtinian conception of dialogism, knowledge production requires at least two interlocutors engaged in a dialogical (thesis, antithesis, and synthesis) interaction (Bakhtin, 1984; Holquist, 2002). Following the concept of currere, Sawyer and Norris (2013) highlighted the need for *“bracketing in” the voices* instead of bracketing them out to achieve a supposed objectivity. In duoethnography, subjectivity and personal epistemology become means of deeper understanding of phenomena and their context. Following “bracketing in”, the *self becomes the research site* in its specific socio-cultural context. The next critical tenet is the *(re)storying of self and other*. Duoethnography, by inviting the other in a dialogic interaction, helps participants unearth their untold stories and cross-pollinate their narratives. By doing so, “The study can create a capacity for future change and deepening of the restorying process.” (Sawyer & Norris, 2013, p. 16). A prerequisite for achieving this is the next tenet, to *quest(ion) not hero/victim*: “If duoethnographers enter into their research and writing with fixed ideas, the result will not be dialogic. The emphasis is on the “quest,” the questioning, because the conversation with the other should change one’s personal stories in some way.” (Sawyer & Norris, 2013, p. 17) Duoethnography celebrates a *fluid, recursive, layered Identity*. Nevertheless, instances of this dynamic nature of the researchers’ identities can be captured in the flux of their dialogic interaction. By promoting a democratization of the research process, duoethnography is also premised on a social constructionist epistemological view, where understandings are not merely discovered*: Meanings are created, exposed and transformed.* As such, duoethnographies are *emergent, not prescriptive* keeping in this way the method open, creative, and playful. Sawyer and Norris (2013) also highlighted the need for a critical dialogic frame, recognizing the existing power differences among the duoethnographers. The *place,* the *literature*, and the *readers* are all also acknowledged as active interlocutors and coparticipants in a duoethnographic dialogue. Finally, a quite critical tenet identified by Sawyer and Norris (2013) was that of the *difference as heurism*: “In duoethnography, contrast and difference are viewed as strengths, not deficits. Duoethnography does not seek universals.” (p. 21).

### A Special Focus on the Difference

Sawyer and Norris (2016), as the first developers of duoethnography, tried to avoid being prescriptive and instead encouraged duoethnographers to evolve their practice and develop their own living versions of duoethnography. Following the above methodological framework, our conception of duoethnography is that of two (at least) auto-ethnographers constantly engaged in a mutually transformative dialogue in such a way as for the resulting research entanglement (both as a process and as an outcome) to be more than the sum of its constituent parts. Sawyer and Norris (2013) urge researchers to not neglect and instead focus on and take advantage of the crucial differences between the intersecting narratives so as for the participating actors to reconceptualize what they think they know about the phenomenon under study, enrich their personal perspectives, and gain new knowledge and insights (Nelson & Phillips, 2019). Duoethnography “examines how different individuals give both similar and different meanings to a shared phenomenon” (Sawyer & Norris, 2013, p. 21) in a process that “should question the meanings about and invite reconceptualization of that past. The Other in the research is invited in order to bring new insights into the old stories.” (Breault, 2016, p. 3) Therefore, duoethnography requires “at least two practitioners working together in tandem in a dialogic format which emphasizes differences in perception between these inquirers.” (Sawyer & Norris, 2016, p. 3).

## Chara and Alexis in a Reflective Dialogue: a Duoethnography

A year has passed since the offering of this program and the beginning of writing this paper. We thought that engaging in an evocative and performative dialogue, in the here and now of the present time, will be a more trustworthy way to communicate our lived experience then, now, and in the between. So, we met, we recalled, we discussed, and we let the interaction drive our thoughts. Such an approach “reframe research from an embodied vantage point: not from the outside in (etic) –from an external, abstract perspective– but from the inside out (emic).” (Sawyer & Norris, 2013, p. 1) Duoethnography is unique in that it emphasizes the researchers and their interacting narratives as the research location (Breault, 2016). In a duoethnography, two researchers collaborate to critically analyze and challenge their interpretations of the social phenomena under study. Researchers want to promote heteroglossia (Bakhtin, 1984), as a multivoiced and critical tension between them (Sawyer & Norris, 2015). As Sawyer and Norris point out (2015), “we sought to turn the inquiry lens on ourselves, not as the topic, but as the site of an archeological examination of the formation of our beliefs, values, and ways of knowing” (p. 1) Duoethnographers participate in dialogical cycles of interpretation that incorporate the investigation of cultural artifacts from their personal histories as well as the expression of fresh emerging viewpoints and ideas by revisiting their lived experiences (Sawyer & Norris, 2013). As they work together to create a polyphonic text, they look for converging as well as diverting viewpoints. The duoethnography approach offers us a new way “to understand ourselves and our project, reflexively considering how both affect one another.” (Wiant Cummins & Brannon, 2022, p. 87) In this context, our dialogue as it emerged in vivo follows.

*Alexis:* While I revisit the online digital traces of our program, in the asynchronous e-class platform we utilized to support our weekly synchronous zoom meetings, intense feelings flood me, and I experience a kind of nostalgia. I recall a journey, an adventure, and a hard effort to realize, design, develop and implement the whole program. A demanding adventure in which, thankfully, I was not alone. A route we co-created and co-traversed together Chara. Now I realize that our storytelling lab was from the very beginning process-oriented, a living duoethnography between you and me, from the very inception of the program two years ago. Maybe this is because life is always a duoethnography, or a collective ethnography, a constant dialogue between living entities. It took almost a year for this program to fully blossom, from its inception to its first pilot offering. I recall now how emotionally rich, appreciative, and meaningful those two last synchronous meetings were in the series of the twelve weeks program course. The two final story circles were so emotionally intense and so enriching. This thrills me. They say it takes two to tango. And now I realize why a duoethnographic experimentation, like this one, is the most appropriate means to reflect back and communicate our lived experience in an isomorphic way. Isomorphic to the project’s inception, design, development, and implementation.

*Chara:* Trying to recall the beginning of this journey there is one thing that keeps coming to my mind: developing a program like this was a big challenge for us. It was the first time that we were working together as co-facilitators, so we have to work out our collaboration. And the idea behind this project was not something easy to communicate and explain to the stakeholders of AIA and the intended audience. When Alexis told me his idea about a digital storytelling workshop, it reminded me of my efforts as a teacher working with students. Fortunately, I had used digital storytelling before with students (adolescents). To me, it was an activity that combines creativity with fun time and is experienced as meaningful and engaging. So, for me, it was obvious that it would be a helpful tool for practitioners working with human systems (our targeted audience). On the other hand, as my previous experience with digital storytelling was only with individuals working alone, I thought that doing DST in a group context would be a far more challenging endeavor, while potentially more powerful and enriching… But in any case, not an easy task for us, I thought at first. As we started discussing this project, ideas came along, and the program began to take a more concrete shape and form. Each of us put ideas that inspired us both and the whole project came to be a process of creating together something more and more fulfilling. Especially for me, it allowed me to build a bridge between my two professional identities in transition, as a psychotherapist/group facilitator on one hand, and as a teacher and educator previously. At that time, I was in a transition as a professional, and the fact that I could utilize ideas from both practices was a relief and a part of my personal, inner, attempt for integrating them both.

Offering the program under the umbrella of the Athenian Institute of Anthropos (AIA), a historical and well-respected center in Greece, was also a great challenge for me. The trust Petros Polychronis (the director of AIA at that time) showed us was a big burden and a great blessing at the same time… And the fact that it was a period when we discovered that he was terminally ill, made this work and our meetings with him even more flourishing, while there was sadness and difficulty in the air. At the same time, on another level, COVID-19 came across and made us all anxious, worried, even scared sometimes, and definitely in front of a computer screen!

*Alexis*: End of an era, I would say. Time seems to pass so fast. The perfect time for Janus, the ancient Roman God of beginnings, gates, transitions, and endings. He is usually portrayed as having two faces, one facing what is left behind, and one facing what follows, what is expected to come. Janus was meant to signify the difficulty of any transition, as we have to farewell something and welcome something else. And today we live in times of dramatic changes, crises followed by more crises without a stop, in a chronotope of everlasting destabilization, in a constant transition. And in this context of glocal turmoils (wars, environmental and financial crises, pandemics), I feel it is an opportunity, a blessing, to engage in this duoethnographic inquiry, a process of reflecting back together on our digital storytelling adventure through a dialogic narrative experimentation. An opportunity to revisit our lived experience in this process-oriented and appreciative storytelling lab we co-dreamed, co-developed, and co-facilitated. Just one year has passed since its first offering, but to me, it seems that a whole era has passed away. The director of the Athenian Institute of Anthropos, the systemic practice center that hosted our program is no longer with us to embrace our dreams and offer us his precious advice. Petros Polychronis, a child psychiatrist, therapist, teacher, appreciative practitioner, and systems thinker, was always an invaluable source of inspiration for us and our work, for disseminating the systems view of life through our group storytelling project. I feel so honored and grateful to be given this opportunity, to live in this specific chronotope and to be lucky enough to have Petros Polychronis as a therapist and teacher…

*Chara:* As far as it concerns the designing and delivery of this program, I think that there were a lot of challenges at different levels: each of us had our expectations, fears, and goals, and we also had to make an effort to build a good pair as facilitators. There were technical issues we had to face, as it was not obvious how we would be able to train participants in the use of the necessary digital tools, since they did not have the same level of digital literacy. Also, it was the first time that a program like this was offered, an integrative program that tries to make use of digital storytelling while building a group that on the whole level it develops a collective meta-narrative, a group story. Offering such a complex and sophisticated program to professional practitioners was not easy for me. It looked like having to climb a whole mountain. How to explain to them, as they had no previous knowledge about that, what they were going to learn and practice in this program? On the other hand, the same challenges made this whole period quite creative and exciting! I remember me creating my own digital story to introduce myself to the group and to provide them with an idea about what a digital storytelling artifact looks like… Developing this first digital story inside a relationship, as Alexis was my audience and provided me authentic feedback, was a quite different experience compared to sharing it with the whole group. I remember discussing when it would be the right moment for us to share with the group our introductory digital stories and for me this was a great issue and challenge since I felt that I would prefer to connect with them before in order to feel safe to share. That process and my feelings proved to be invaluable information I would use to understand members’ feelings and attitudes and also indicators about where we were as a group, and how ready we were to share more intimate things.

*Alexis*: As I listen to your reflection Chara, I will focus on one point. It seems to me that what you say confirms what we know from existential psychotherapy theory (specifically Victor Frankle’s approach) and systems thinking. Human systems, as purpose and meaning creators, often seem to have a knowledge about the future they are going to create, before the actual way or the details of this future can be realized or even imagined. There is nothing magical in this; in such case, the actual knowledge is still embodied within us but not “verbal enough” to be able to communicate it, even to ourselves. I recall the “incubation” period of our project. We were dreaming, designing, and shaping it. And despite the stress of the first time, of the unchartered waters we are heading to, and despite a sense of big responsibility on our shoulders due to what AIA institute meant to us, deep inside, I always felt confident about the result. And this is in contrast to the fact that, usually, I am a person with many insecurities. Nevertheless, I always felt quite confident about this storytelling project we were preparing like having a deep unconscious knowledge about it. Today, I feel I can clearly communicate the fundamental pillars of this project: (a) group work and facilitating dense meaningful interactions among participants to allow for a coherent group to emerge, (b) scaffolding multimodal narrative practice in organized activities to allow personal stories to slowly unearth in the context of (c) an appreciative and inclusive culture. These also happen to be the fundamental pillars in my professional systemic practice as well, either in the psychotherapeutic or educational settings I work. So somehow this theoretical grounding of our project came naturally to me. I believe that these three pillars, coupled with the use of modern technologies and digital tools, created the optimal conditions for participants to develop as individuals and for the group to flourish as a whole. And I feel so grateful that we had the opportunity to experience and witness this unique transformative process during our weekly lab meetings. I feel that what we witnessed during, and especially in concluding, this program surpassed by far our expectations. It is this feeling of genuine human connection, the moment you are in a group, and you feel so connected as like your hearts are beating together in synchrony, and your mirror neurons are firing in concert, the eyes glowing, and you experience a pause of the time, an evocative silence full of warmth that lasts for a few moments but it is experienced so intensively. And then, this experience will become an embodied deep knowledge for the rest of your life, to accompany you in every subsequent group process you are going to facilitate, empowering you without even realizing it in every difficulty you are going to face. This is how I experienced that feeling of deep connection, and I am grateful to Chara that due to our duoethnographic experimentation, I had this opportunity to somehow put it in words.

*Chara:* There is one thing I like to highlight, our work as collaborators at different levels. As facilitators, I think we managed to focus both on the individuals and the whole group level. Participants’ weekly reflections on the asynchronous platform proved to be so important for building group cohesiveness and for us to follow their ideas and feelings on a personal level. At the same time, we were able to give them back every week a meta-narrative, a synthesis of what we heard the group telling us through their personal reflections. We also utilized their multimodal reflections as building blocks for creating a new digital story, the weekly group meta-narrative. Our feedback was well received by the participants and as the sessions progressed some members were reflecting back on what the group is telling through these collective meta-narratives.

It became evident through their week-by-week individual storytelling reflections that the group process was slowly and steadily evolving. Gradually, others’ voices became external positions in the individuals’ inner dialogues and their storytelling reflections progressively became more and more multimodal and polyphonic. Occasionally, weekly storytelling reflections seemed to address particular audiences, either specific members of the group, or the group as a whole. Meanwhile, these reflections were a place for experimentation with digital tools and non-textual ways of expression. The first weekly reflection assigned to the group was a textual one, and after that, week by week, reflections became more multimodal, combining text with a photo in the second week, a drawing with text later, then with a comic, and then with personal photos. Later, voice narration and background music were added to the already developed set of modalities to form short but fully developed digital stories. It is so amazing to study after the end of the program all those progressively developed digital storytelling artifacts and acknowledge all those different media, ideas, and stories that were shared during these twelve weeks. All these digital storytelling traces left in the program’s asynchronous platform (and always accessible to the participants as a record of their collective history), along with the dialogues unfolded between group members at different levels and interactive media, constitute an invaluable manifestation of the group’s gradual development and evolution, a collective history of becoming.

At the same time, my relationship with Alexis also developed, as an ongoing dialogue, negotiating roles and trying to collaborate constructively. I remember that for me it was a very demanding period not only professionally but also for my family as some unpredictable, serious incidents disturbed me. The way that Alexis supported me and our collaboration was a relief for me. I think that the most important part of our work was our co-operation. As a sub-system in the system of the whole community we were creating, as the facilitating pair, we had a positive effect on the participants. In our epistemology, second-order cybernetics, the observer is always part of the system that observes. Effectively, I feel that this principle was manifested as a co-evolution between the development of our pair and the development of the participants’ group. At this point, I feel that the most important thing for me was the trust I felt for my co-facilitator, a product of the work and sharing that took place between us. This made it easy for me to feel safe to share my thoughts, and feelings with Alexis, and to allow for the differences to be easily communicated and acknowledged. From my point of view, it was not just an accident, a serendipitous coincidence, that our professional relationship, and the way we were communicating and collaborating, were highlighted by the participants at the end of our program as so important. In their words, our professional relationship became a model for their own partnerships in the future.

As I recall our concluding sessions, I have to mention the final story circle which was so impressive to me. A big surprise since I couldn’t think it would be so touching, with so intense emotions and words that had a deep impact on all of us. Group members, now well-trained digital storytellers, shared their personal stories with courage, dignity, authenticity, respect, and care for their self-disclosure and for accepting and receiving what other storytellers brought into the circle. As I revisit members’ reflections after the story circle, I have kept some phrases that have a special meaning for me. So, I made a synthesis of them, a meta-narrative: “these stories were little priceless gifts that I offer to and receive from the group”, “in really dark periods”, “stories different but also similar”, “with parallel routes”, “which turn out that have some elements in common”, and “which are like journeys that connect us with our dreams” as “life finds always a way to move forward”…

Before the end of this journey, I was very anxious about what the members took with them, and whether the program was really helpful and meaningful… A lot of worries, but the final story circle relieved me, not cognitively, as this part requires another kind of processing, like the one that takes place right now through this article writing and duoethnographic experimentation, but emotionally, unconsciously, whatever words can put someone in my mouth, in a non-explicit way, I could say…

What was really important for me after the conclusion of the program was the efforts that some members did to utilize the program or some parts of it in their own work with communities. One member applied this approach to digital storytelling (informed by systems thinking and appreciative inquiry) in a short program for empowering a group of teachers. Two other members utilized some tools and activities of our program in their work with adolescents. Another member had the idea of making a fairy tale inspired by her digital story that would be published in print. In the most recent effort, a participant that works as a psychotherapist, being inspired by her work in the program, helped a young woman on the Autism spectrum to write her story and now she is ready to publish her book! For me, all these spin-offs inspired by our work are something quite rewarding and help me realize that this program “is what it is” as a member once wrote in one of their reflections (quoted from an Erich Fields’ poem) and this is enough.

*Alexis*: I am thinking of the rhizome metaphor: An invisible complex network of interconnections with our personal stories as nodes. A rhizome that is always in the becoming and shaped by the unique ways each one of us narrates our own lived existence. A kind of a collective storified unconsciousness. When communicated in the context of an appreciative group, our stories look so different and at the same time so similar. We share common patterns, fears, hopes, and enabling or constraining narratives. And I feel that in the virtual group mirror they co-create, storyteller participants can recognize their hidden (but still defining them) narratives, and identify new ways to re-author their personal stories. It has been said that there are no truths, only stories. Under a social constructionism epistemological view, the way we narrate our existence does not simply represent our reality; it constantly (re)creates that reality. Revisiting now, after a year, the digital footprints of our project in the e-class environment we used, a rich set of digital storytelling artifacts have been left there to echo our collective journey. It is still available to the whole group, an online museum to remind everyone of the virtual rhizome we co-created, an evocative collection full of memories and emotions. However, I now realize that I had not revisited this place after the conclusion of the project, and before writing this duoethnography. Life advances constantly without slowing down, chapters close, and new open up all the time, the practicalities of modern life, maybe a fast-forward mode of living up. And I recall our discussion Chara, and your point about how important it is for the emotional connection experienced in a group work session, for the emotions to be further processed and integrated cognitively. And reflecting back on our practice and lived experience and actively expressing it in words by writing a duoethnography, serves this cognitive integration. It establishes a bridge between the present and the past lived experience regarding this project, and not only. It adds elements of cognitive processing to the emotional experience. It helps us realize how important it is to share our reflections with the entire group and to call for a collective autoethnography project. If not now, maybe in the next chapter of our life story. Thank you, Chara, for the idea. As one of our teachers in systems practice points out, *I become smarter when I speak to you*.

## Discussion: Focusing on the Difference

In the famous words of Gregory Bateson, it is *the difference that makes the difference* (Bateson, 1979). As in a duoethnography “spaces of multiplicity are generated by recognizing the differences—not the similarities” (Sawyer & Norris, 2016, p. 8), in this section we will attempt a re-reading of the previous dialogue to highlight the major differences in the two co-evolving narratives. The aim is to reflect on those differences and learn from them.

In opening the conversation, Alexis sets the context of the inquiry in the present time (that of writing the article) providing the rationale for this duoethnography “*life is always a duoethnography, or a collective ethnography, a constant dialogue between living entities*”. Reading this part of the conversation retrospectively, we can spot what Breault (2016) identifies as the risk of mere theory confirmation or, in other words, narrating a success story: “*It took almost a year for this program to fully blossom, from its inception to its first pilot offering. I recall now how emotionally rich, appreciative, and meaningful those two last synchronous meetings were in the series of the twelve weeks program course.*” Maybe this is an inherent tendency in academic writing to overemphasize the strengths and cover the difficulties faced in a research project. As Nobel laureate physicist Richard Feynman (1965) argues in his Nobel award lecture opening, “We have a habit in writing articles published in scientific journals to make the work as finished as possible, to cover all the tracks, to not worry about the blind alleys or to describe how you had the wrong idea first, and so on.” Nevertheless, what seems to be more anchored in the actual lived experience of Alexis is the recalling of the two final story sharing circles and the intense emotional experiences associated with: “*The two final story circles were so emotionally intense and so enriching. This thrills me.*”

On the other hand, in the next dialogue turn, Chara focuses more on the personal challenges, difficulties, and practicalities the authors faced both as human beings and practitioners. Chara speaks about the time when the program was delivered (about a year before writing this article). Chara seems to wonder: *How our design came into existence and how practically unfolded? What were the practical challenges we faced? And how and in what ways has this project helped us shape our professional identities?* Next, Alexis continues the ‘success story’ narrative (“I feel it is an opportunity, a blessing, to engage in this duoethnographic inquiry”) setting the time again in the here and the now of the dialogue (one year after concluding this program) and referring to the dramatic changes that followed (the passing away of Petros Polychronis, an inspirational figure for both practitioners).

In the next dialogue turn, Chara keeps talking in a more personal, practice-oriented tone, describing practicalities, and providing specific details of challenges we faced: “*Also, it was the first time that a program like this was offered, an integrative program that tries to make use of digital storytelling while building a group that on the whole level it develops a collective meta-narrative, a group story. Offering such a complex and sophisticated program to professional practitioners was not easy for me.*” While, until this point, Alexis seems to adopt a more ‘epic’, grand-narrative style, Chara seems to follow a more ‘novel’ narrative style, in Bakhtinian genre-theory terms (Bakhtin, 1984), describing how she experienced the program in a multivoiced situated fashion: “Bakhtin conceives existence as the kind of book we call a novel, or more accurately as many novels… for all of us write our own such text, a text that is then called our life.” (Holquist, 2002, p. 30).

In the following turn, Alexis continues to focus on the rationale of the program. He provides specific implementation details having always the ‘grand picture’ in mind “*It seems to me that what you say confirms what we know from existential psychotherapy theory (specifically Victor Frankle’s approach) and systems thinking. Human systems, as purpose and meaning creators, often seem to have a knowledge about the future they are going to create, before the actual way or the details of this future can be realized or even imagined. There is nothing magical in this; in such case, the actual knowledge is still embodied within us but not ‘verbal enough’ to be able to communicate it, even to ourselves.*” It could be that Alexis participates in the dialogue having an imagined audience (for this article) in mind and, therefore, trying to explain the rationale of the whole program. Such an approach maybe is vulnerable to what Breault (2016) identifies as the risk of independent conclusions simply being reaffirmed. Nevertheless, realizing the meaning of the parts in the context of the whole they co-create is a fundamental premise in system thinking, and the whole program was always about a holistic, community-oriented view of digital storytelling.

In the next turn, a shift happens, as Chara seems to bring now the “grand picture” in mind while focusing on the detailed description of the program’s rationale: “As facilitators, I think we managed to focus both on the individuals and the whole group level. Participants’ weekly reflections on the asynchronous platform proved to be so important for building group cohesiveness and for us to follow their ideas and feelings on a personal level. At the same time, we were able to give them back every week a meta-narrative, a synthesis of what we heard the group telling us through their personal reflections. We also utilized their multimodal reflections as building blocks for creating a new digital story, the weekly group meta-narrative.” However, Chara continues by assuming back the more emic, personal, perspective: “I remember that for me it was a very demanding period not only professionally but also for my family as some unpredictable, serious incidents disturbed me. The way that Alexis supported me and our collaboration was a relief for me. I think that the most important part of our work was our co-operation. As a sub-system in the system of the whole community we were creating, as the facilitating pair, we had a positive effect on the participants.” But at this point, it seems that Chara now combines the personal *emic* view with theory development and epistemology (going from the inside out): “In our epistemology, second-order cybernetics, the observer is always part of the system that observes. Effectively, I feel that this principle was manifested as a co-evolution between the development of our pair and the development of the participants’ group. … From my point of view, it was not just an accident, a serendipitous coincidence, that our professional relationship, and the way we were communicating and collaborating, were highlighted by the participants at the end of our program as so important. In their words, our professional relationship became a model for their own partnerships in the future.”

In the concluding turn, Alexis continues to reflect on the specific digital storytelling program, while on a different level, it seems to reflect on the very epistemology of duoethnography: “I am thinking of the rhizome metaphor: An invisible complex network of interconnections with our personal stories as nodes. A rhizome that is always in the becoming and shaped by the unique ways each one of us narrates our own lived existence. A kind of collective storified unconsciousness. When communicated in the context of an appreciative group, our stories look so different and at the same time so similar. We share common patterns, fears, hopes, and enabling or constraining narratives. And I feel that in the virtual group mirror they co-create, storyteller participants can recognize their hidden (but still defining them) narratives and identify new ways to re-author their personal stories.”

Duoethnography examines how participants give both similar and different meanings to a shared event (Sawyer & Norris, 2009). By focusing on the differences between us, we identified an interplay and an interweaving between the local and the global, between the emic and the etic, between the personal story and the grand narrative, between the novel and the epic, and between the actual other and an imagined audience. We also identified an isomorphism between the process of duoethnography as a research practice and the described relational community digital storytelling program. Focusing on the different perspectives and their interplay is crucial for gaining new insights and allowing new interpretations to emerge since existence is “the event of co-being; it is a vast web of interconnections each and all of which are linked as participants in an event whose totality is so immense that no single one of us can ever know it.” (Holquist, 2002, p. 41).

## Concluding Thoughts: the Difference That Makes the Difference

From its first inception as a conference presentation, the title of this article was *Relational, appreciative, and process-oriented digital storytelling.* The second part of the title (*A duoethnography*) was added later to reflect the research approach we employed to deepen our inquiry. We thought that these three qualifiers (relational, appreciative, and process-oriented) would accurately describe the main pillars of our practice as were manifested in the specific digital storytelling program and our overall professional praxis as psychotherapists and systems thinking practitioners. We now realize that also duoethnography as a research method happens to be relational, appreciative, and process-oriented. Duoethnography is *relational* by design as it engages two (at least) interlocutors in a deep dialogical inquiry. It is also *process-oriented* as it is grounded on the concept of *currere*. Duoethnography is also an *appreciative* practice although this quality is not yet articulated in the existing set of tenets. This is because to engage in a transformative dialogue with another human being you need first of all to acknowledge the value of the other person and recognize their difference as something you can learn from.

And at this point, another critical qualifier, the *difference*, comes into play*.* Focusing on the difference is a fundamental tenet of duoethnography, a driving force for personal and collective transformation. The difference is something that we, as practitioners and researchers working with human systems, should not be afraid of; instead, it is something we should embrace and take advantage of. “Every discovery has a painful and a joyful side: painful while struggling with a new insight; joyful, when this insight is gained.” (Foerster, 1984, p. 42) Acknowledging the difference as a valuable heurism was a transformative shift for us that this duoethnography catalyzed. In Bakhtinian thought, the establishment of a relationship between two persons occupying different points of view is critical for creating meaning:

You can see things behind my back that I cannot see, and I can see things behind your back that are denied to your vision. We are both doing essentially the same thing, but from different places: although we are in the same event, that event is different for each of us. (Holquist, 2002, p. 22)

In duoethnography, two researchers collaborate to dialogically question and challenge their interpretations of a shared event (Sawyer & Norris, 2009). This is also the case with our proposed relational, appreciative, and process-oriented digital storytelling program where the members of a group collaborate to enrich their personal stories and develop as a community. As von Foerster (1984) points out, “perceiving is doing, and if I don’t see I am blind, I am blind; but if I see I am blind, I see.” (p. 43) And to see whether I am blind I need at least another person with a different point of view to shed light on my own blind spots.

## Data Availability

The authors confirm that all data generated or analyzed during this study are included in this published article.

## Appendix

Participant 1: “This is about creating a connection with own self and with the group. The medium matters: it helps you author your story and reframe your previous experience. It is a kind of memory recording that helps you observe your evolution, what was your beginning, and where you have arrived. It is a way of expressing feelings, and thoughts, a way of sharing. When you develop a digital story and you expose yourself, this is so authentic that there is no space left for criticism. Technology and people are coupled to create. Each member offers its own skills, and a collective result emerges.”

Participant 2: “It is relating to myself, to the others, and to the outer world evolving around me, while having the freedom to select when, what, and how much I am going to share. Creativity, co-creation, and exploration. It helped me to rediscover sides of myself that I had forgotten long before.”

Participant 3: “A journey into a world where I am the creator.”

Participant 4: “It was for me a way of sharing, connecting, and telling stories that have made me more human. It was a presentation of myself to a group, through digital media.”

Participant 5: “It is image, communication, music. Transport in space and time. Lasting in space and time. Creates memories, and lived experiences, on a collective or individual level. It can be serious or funny. A route through human lives and ideas. A reflective journey.”

Participant 6: “The program was an oasis during the week. I learned how to dive into the depths of the digital. The group became a safe place to rest upon and share. The facilitators were there, ready to provide answers to every question, to honor all voices and emotions with respect and care.”

Participant 7: “What digital storytelling means to me? it is relating. This is the first spontaneous answer that comes to me. I could stop writing here. Yes, I definitely got knowledge of how to use specific storytelling tools. I also got guidance, and I felt cared for. That's why tools are not the most important part for me. I came here at a time in my life I was quite busy and I turned to this to do something more ‘relaxed’. It turned out to be one of the most rewarding things I've ever done. I made connections with other colleagues and wonderful people. I also reconnected with aspects of myself that for some reason I neglected to bring to the foreground: my creative self, my writing self, my determined self.”

Participant 8: “I communicate a story, my story, in an original and modern way to people who listen to me appreciatively.”

Participant 9: “Making and sharing stories that connect people with the help of digital technologies, the use of colors and sounds, drawings, animation, and other creativity tools.”

Participant 10: “For me, digital storytelling is a medium. Medium for expression, for communication, for relating with each other. The focus was never on technology; technology was just the means to be heard in a qualitatively different way. But when we manage all together to create a whole, the beauty of sharing becomes magic, the magic of connection. And it is this connection that makes us feel humane. Our stories make us more humane.”
